# Noninvasive ventilation with a helmet in patients with acute respiratory failure caused by chest trauma: a randomized controlled trial

**DOI:** 10.1038/s41598-020-78607-5

**Published:** 2020-12-08

**Authors:** Qi Liu, Mengtian Shan, Hailong Zhu, Jianliang Cao, Rongchang Chen

**Affiliations:** 1 Emergency Intensive Care Ward, The First Affiliated Hospital of Zhengzhou University, Zhengzhou University, Zhengzhou, Henan China; 2 Shenzhen Institute of Respiratory Diseases, Shenzhen People’s Hospital, Shenzhen, Guangdong China; 3 State Key Laboratory of Respiratory Disease, Guangzhou Institute of Respiratory Diseases, First Affiliated Hospital of Guangzhou Medical University, Guangzhou, Guangdong China

**Keywords:** Randomized controlled trials, Trauma

## Abstract

Noninvasive ventilation (NIV) is beneficial in acute respiratory failure (ARF) caused by chest trauma; however, NIV-related complications affect the efficacy. We evaluated whether NIV with helmet decreases the incidence of complications and improves its effects in a single center. Patients with ARF after chest trauma were randomized to receive NIV with helmet or face mask. The primary outcome was the rate of NIV-related complications. Secondary outcomes were PaO_2_/FiO_2_, patient’s tolerance, intubation rate, length of intensive care unit (ICU) stay, and ICU mortality. The trial was terminated early after an interim analysis with 59 patients. The incidence of complications was lower in the helmet group [10% (3/29) vs 43% (13/30), *P* = 0.004], and PaO_2_/FiO_2_s were higher at 1 h and at the end of NIV (253.14 ± 64.74 mmHg vs 216.06 ± 43.86 mmHg, 277.07 ± 84.89 mmHg vs 225.81 ± 63.64 mmHg, *P* = 0.013 and 0.012) compared with them in face mask group. More patients reported excellent tolerance of the helmet vs face mask after 4 h of NIV [83% (24/29) vs 47% (14/30), *P* = 0.004] and at the end of NIV [69% (20/29) vs 30% (9/30), *P* = 0.03]. Differences in intubation rate, ICU stay, and mortality were non-significant (*P* = 0.612, 0.100, 1.000, respectively). NIV with helmet decreased NIV-related complications, increased PaO_2_/FiO_2_, and improved tolerance compared with NIV with face mask in patients with chest trauma.

**Trial registration:** Registered in the Chinese Clinical Trial Registry (ChiCTR1900025915), a WHO International Clinical Trials Registry Platform (http://www.chictr.org.cn/searchprojen.aspx).

## Introduction

Chest injury is common in patients with trauma^1^ and accounts for 25–40% of all trauma-related fatalities^2,3^. Acute respiratory failure (ARF) caused by pulmonary contusion, rib fractures, pneumothorax, and hemothorax occur frequently, despite the use of oxygen therapy and regional analgesia^4–6^. Post-traumatic ARF within 72 h is associated with a high mortality rate^7,8^, and as a result, patients require rapid and efficient ventilatory management. Endotracheal intubation and invasive ventilation is performed in 23–75% of patients with chest trauma^9^; however, these procedures are associated with increased complications, and prolonged ventilation and hospitalization times^9–12^.

Recent studies demonstrated that patients with ARF after chest trauma responded favorably to noninvasive ventilation (NIV)^13–16^, which could reduce the need for intubation, the incidence of pneumonia, and mortality rates^10,17,18^. Nonetheless, complications and discomfort associated with NIV delivered via a face mask are common and result in NIV failure rates of up to 14.8% in patients with chest trauma^19^.

Chest trauma itself causes pain and patient irritability, and may be associated with facial injuries, which interfere with the patient’s ability to cooperate with NIV with a mask. A recently-developed helmet has been used effectively to deliver NIV in select patients^20–23^. The helmet does not touch the patient’s face, and allows patients to eat and talk, which improves tolerance and prevents skin lesions^22^. We hypothesized that NIV with the helmet might reduce NIV-related complications, be easier to tolerate (especially for patients with mild facial injuries), and improve the effects of NIV in patients with chest trauma. However, to date, there are no data describing this important issue; therefore, we performed a randomized controlled trial (RCT) to investigate our hypothesis.

## Methods

This single-center trial was registered in the Chinese Clinical Trial Registry (ChiCTR1900025915, 14/09/2019) and was approved by the Ethics Committee of the First Affiliated Hospital of Zhengzhou University (No: SS-2019-002). This teaching hospital has 6500 beds and a provincial trauma center with 20 intensive care beds for patients with severe trauma. All patient management was performed in accordance with relevant guidelines and regulations^15,24,25^. Consecutive patients admitted to the emergency intensive care unit (ICU) of this hospital from 1 September 2018 to 1 July 2019 were screened according to the eligibility criteria. Informed consent was obtained from all included patients or their relatives.

### Participants

The inclusion criteria were as follows: (1) Older than 18 years; (2) within the first 72 h after chest trauma; (3) trauma confirmed by imaging; (4) moderate to severe hypoxemic respiratory failure, which was defined as the ratio of the partial pressure of oxygen in arterial blood to the fraction of inspired oxygen (PaO_2_/FiO_2_) < 200 mmHg while receiving standard oxygen therapy with an oxygen flow rate ≥ 10 L/min via a face mask at least for 15 min; and (5) informed consent signed by the patient or a close relative.

Patients meeting any of the following criteria were excluded: (1) impending cardiopulmonary arrest or the need for emergency intubation; (2) unable to accept NIV treatment because of decreased consciousness (Glasgow coma scale score ≤ 11)^26–28^; (3) contraindications for NIV (active gastrointestinal bleeding, upper airway obstruction, or severe hemodynamic instability); (4) severe facial trauma with pneumocephalus or involving a sinus, skull base fracture, or orbital fracture; (5) cervical injury; (6) increased intracranial pressure; and (7) declined to provide signed informed consent.

### Randomization and masking

We randomized patients in a 1:1 ratio using a computer random number generator. The random numbers were generated and secured in sequentially numbered, opaque, sealed envelopes kept by the head nurse. After determining that a potential research participant was eligible for inclusion according to the inclusion and exclusion criteria, the researchers obtained a sealed envelope from the head nurse, and the patient randomly received a helmet (odd number) or a face mask (even number) according to the disclosed random number. Data were recorded in the medical record system and the pre-piloted forms by the nurses and therapist who were blinded to the randomization. The study investigators collected the data, and the nurses and related medical staff managed patients without discrimination.

### Basic treatment protocol

Basic treatment constituted regional analgesia (mainly epidural analgesia with fentanyl plus bupivacaine) unless contraindicated. The efficacy of analgesia was measured using a visual analog scale. Contraindications for regional analgesia were skin injury or infection at the puncture site, or coagulopathy. When epidural administration was impossible, patient-controlled intravenous analgesia was used. Both groups were treated with similar fluid management protocols, and patients’ respiratory and clinical status were monitored during NIV.

### Ventilation protocol

Patients in both groups received noninvasive ventilation (Servo-I; Maquet, Rastatt, Germany) in the pressure support (PS) mode. Patients in the intervention group received NIV with a suitably-sized helmet (dual-connector for breathing circuits) (CaStar; StarMed, Mirandola, Italy), which was made of transparent latex-free polyvinyl chloride and which was secured via two armpit braces to four hooks on a plastic ring. A helmet without armpit braces, with an annular openable ring placed underneath an inflatable cushion, could be used for patients with rib fractures, if necessary. Patients in the control group received NIV with a suitably-sized air-filled oronasal face mask (Tuoren Medical Instrument Co., Xinxiang, China) to ensure a tight and comfortable seal. PS was initially set at 8 cm H_2_O, positive end-expiratory pressure at 5 cm H_2_O, and FiO_2_ at 40%. According to the patient’s clinical symptoms and their percutaneous blood oxygen saturation (SpO_2_), NIV supports were sequentially increased in 1–2-cm H_2_O increments. If respiratory distress and SpO_2_ did not improve, FiO_2_ was progressively increased in 5% increments to achieve an SpO_2_ > 92%. SpO_2_ monitoring was performed strictly according to the equipment manufacturer’s instructions to minimize the impact of factors, such as movement of the patient’s finger to which the monitor was applied or the presence of nail polish, on the accuracy of SpO_2_. In cases of discrepancy between the SpO_2_ reading and a patient’s clinical presentation, arterial blood gas analysis was performed for further confirmation. Short interval disconnections to increase the patient’s tolerance or to clear respiratory secretions were allowed.

### Weaning protocol

NIV support and FiO_2_ were reduced gradually in 1–2 cm H_2_O increments and 5% increments, respectively, if necessary. Weaning from NIV was considered possible when the patient felt dyspnea relief, SpO_2_ was > 92%, PS was decreased to < 8 cm H_2_O, and FiO_2_ was set at 40% with stable hemodynamics.

### Criteria for endotracheal intubation

Patients meeting any of the following criteria were intubated immediately: (1) breathing too weak to trigger the ventilator, or cardiac arrest; (2) severely altered level of consciousness making patients unable to cooperate with NIV; (3) severe hemodynamic instability (defined as mean arterial pressure < 60 mmHg and no response to vasoactive agents); (4) excessive sputum beyond the patient’s expectoration capacity; (5) refractory hypoxemia (SpO_2_ < 85% despite a high oxygen fraction); and (6) unable to tolerate the helmet or face mask. The final decision to intubate was made by consensus among all physicians, excluding the investigators.

### Outcomes

The primary outcome was the rate of complications related to NIV during the ICU stay, which was defined as the ratio of patients who developed any complication to the total number of patients. NIV-related complications included claustrophobia, skin lesions, severe air leakage, eye irritation, gastric distension, and poor tolerance, which were evaluated by nurses and the therapist as part of the daily routine care, and who were blinded to the randomization. Complications were evaluated at 1 h, 4 h, and at the end of NIV, or recorded directly from patients at any time after starting NIV. The secondary outcomes were PaO_2_/FiO_2_, patient tolerance, respiratory rate (evaluated at 1 h, 4 h, and at the end of NIV), intubation rate, duration of NIV, length of ICU stay, and ICU mortality (evaluated at ICU discharge). Skin lesions were defined as a score of at least 1 using the following scale: 0: no lesions, 1: area of redness, 2: moderate skin breakdown, 3: skin ulcer, and 4: skin necrosis, according to a previous study^29^. As air leakage affected the triggering, breathing cycle, and synchrony between the patient and the ventilator, in cases of air leakage, we adjusted the interface and fixation, replaced the helmet or face mask with the appropriate size of interface, and changed the trigger sensitivity. If air leaks still caused an unsuitable PS flow cycle, we changed to noninvasive pressure-controlled ventilation, which we considered to indicate severe air leakage^30–32^. Eye irritation was defined as excessive eye secretions, conjunctival congestion and edema, and any damage to the cornea or iris caused by the interface. Patients’ tolerance was recorded at planned observation time points after beginning the study, and tolerance was evaluated using the following patient tolerance scale: poor, patients try to remove the mask or helmet; moderate, ventilation with a face mask or helmet is successful with suggestions for the patient; good, face mask or helmet use is slightly uncomfortable for the patients, but they want to use it; excellent, complete tolerance^21^. Nurses evaluated complications related only to skin care; clinical indicators such as ICU stay and patients’ tolerance were assessed by doctors and the investigators.

### Statistical analysis

We calculated the sample size based on the primary outcome of the pre-experiment in 16 patients with chest trauma-related ARF. With an expected incidence of complications of 37.5% in the face mask group and 12.5% in the helmet group with 80% power and a two-sided α of 0.05, the required sample size was 88 patients (44 in each group) using PASS 11.0 software (NCSS, Kaysville, UT). An interim analysis was performed at the halfway point in the planned 20-month duration of the study (from 1 September 2018 to 1 May 2020). The predefined NIV discontinuation determinant was a significant (*P* < 0.005) difference in the incidence of complications between the two groups in the interim analysis according to the O'Brien–Fleming method^33^.

We reported our results in accordance with the CONSORT guidelines^34^. All pre-specified analyses were performed by an intention-to-treat analysis. For continuous outcomes, data with a normal distribution were presented as mean ± standard deviation (SD), and these data were analyzed with an independent samples t test; otherwise, the data were reported as median [interquartile range (IQR)], and data were analyzed with the Mann–Whitney U test. For categorical variables, the outcomes were compared using the Chi square or Fisher’s exact test. The time courses for arterial blood gas variables (PaO_2_/FiO_2_, partial pressure of arterial carbon dioxide, and pH) and respiratory rates were compared using two-way analysis of variance for repeated measures within both groups. The level of significance was set at 0.05, and all analyses were performed using SPSS statistical software version 22.0 (IBM Corp., Armonk, NY).

## Results

**Figure 1:**
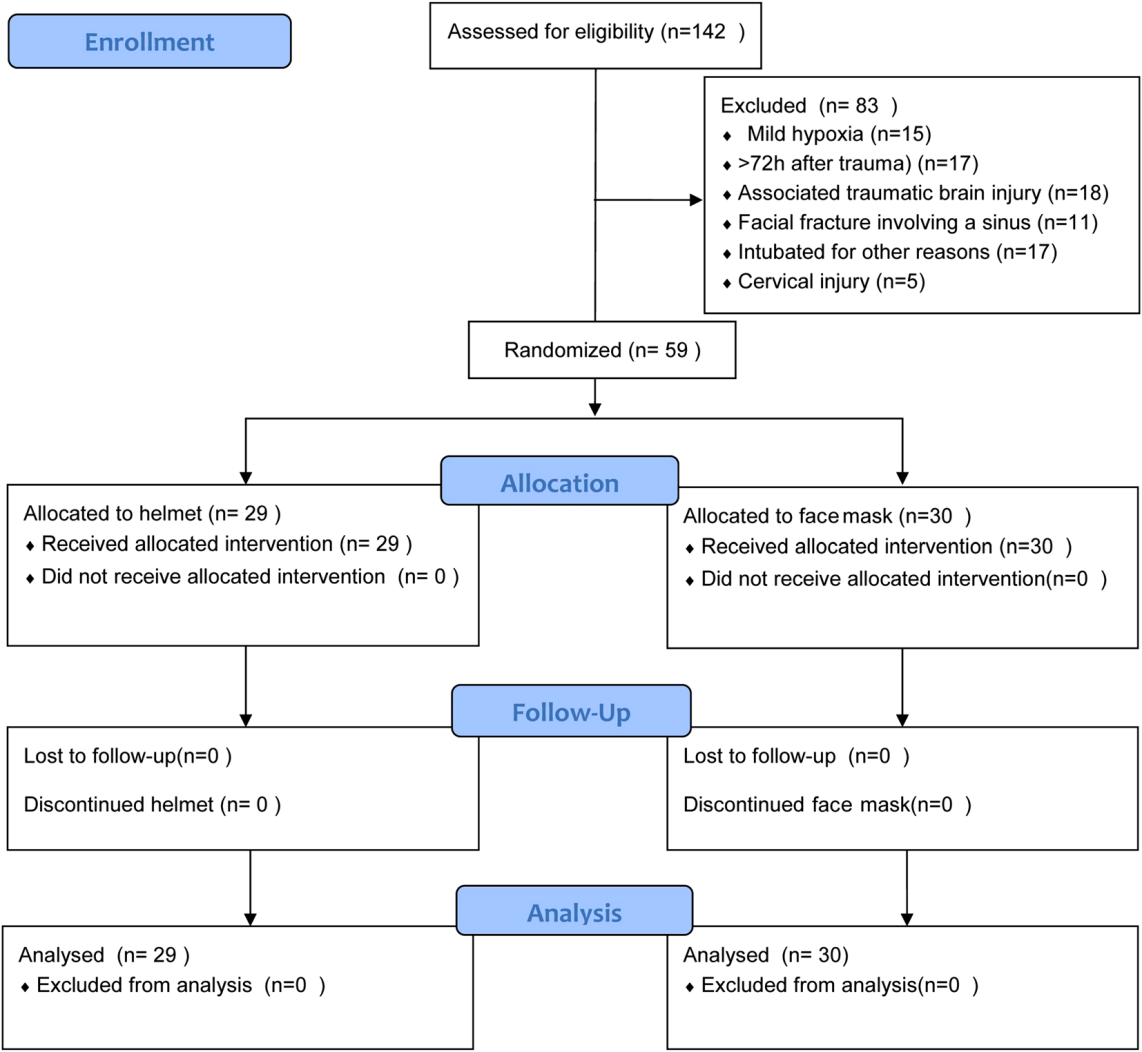
Flow chart of inclusion participants. At the planned time of interim analysis, and the patients included were 29 in helmet group versus 30 in face mask group with *P* = 0.004, which met the early termination criterion (*P* < 0.005) and introduced the sample size in helmet group is an odd number and face mask group is an even number.

From September 2018 to July 2019, 142 patients were screened. After recruiting 59 patients, the lower incidence rate of complications in the helmet group met the early termination criteria for the study compared with the face mask group (*P* = 0.004) in the planned interim analysis after a study duration of 10 months on 1 July 2019 (half of the planned duration of the trial). We finally randomized 29 patients to the helmet group and 30 patients to the face mask group (Fig. 1). All patients completed the study, and their baseline characteristics are shown in Table 1.

**Table 1:** Characteristics of patients at baseline.

	Helmet (n = 29)	Face mask (n = 30)
Age, years	49.24 ± 14.20	49.06 ± 15.90
Male sex	23 (79%)	27 (90%)
**Comorbidities**		
Chronic bronchitis	0 (0%)	1 (3%)
Hypertension	6 (21%)	7 (23%)
Coronary heart disease	2 (7%)	4 (13%)
Cerebral infarction	2 (7%)	3 (10%)
Diabetes mellitus	2 (7%)	4 (13%)
**Mechanism of trauma**		
Vehicle collision	20 (69%)	22 (73%)
Pedestrian traffic injury	4 (14%)	3 (10%)
Thoracic compression	2 (7%)	2 (7%)
Fall	2 (7%)	3 (10%)
Sharp injury	1 (3%)	0 (0%)
**The type of chest trauma**		
Pulmonary contusion	13 (45%)	18 (60%)
Rib fractures	9 (31%)	8 (26%)
Hemothorax	5 (17%)	2 (7%)
Flail chest	2 (7%)	2 (7%)
With slight facial injury	3(10%)	2(7%)
APACHE II, points	11.34 ± 4.83	9.63 ± 3.88
Thoracic AIS, median (IQR) points	4 (3.5–4)	4 (3–4)
ISS, points	23.93 ± 8.87	22.70 ± 8.39
GCS median (IQR) score	15 (15–15)	15 (15–15)
PaO_2_/FiO_2_, mmHg	162.63 ± 25.03	161.94 ± 27.81
PaCO_2_, mmHg	37.91 ± 4.81	37.90 ± 5.25
Arterial pH	7.42 ± 0.05	7.42 ± 0.04
RR, breaths/min	25.48 ± 5.44	23.63 ± 3.76
HR, breaths/min	98.00 ± 19.29	96.67 ± 15.46
SBP at admission, mmHg	123.03 ± 16.43	132.87 ± 18.33
PEEP, median (IQR) cmH_2_O	5 (5–5)	5 (5–5)
PS, median (IQR) cmH_2_O	9 (9–10)	9 (8–9)

*APACHE* Acute Physiology and Chronic Health Evaluation, *AIS* Abbreviated Injury Scale, *ISS* Injury Severity Score, *GCS* glasgow coma scale, *PaO*_*2*_*/FiO*_*2*_ the ratio of partial pressure of oxygen in arterial blood to fraction of inspired oxygen, *PaCO*_*2*_ arterial partial pressure of carbon dioxide, *RR* respiratory rate, *HR* heart rate, *SBP* systolic blood pressure, *PEEP* positive end expiratory pressure, *PS* pressure support, *IQR* interquartile range.

### Effect of NIV with the helmet on the complications rate

The incidence rate of complications related to NIV was lower in the helmet group than in the face mask group [10% (3/29) vs 43% (13/30), respectively; *P* = 0.004] (Table 2). Complications in the helmet group were claustrophobia in two patients and neck skin redness in one patient. In the face mask group, complications were facial skin lesions, air leakage, eye irritation, and gastric distension in 13 patients. None of the patients with facial trauma developed complications in the helmet group, whereas one patient developed complications in the face mask group.

**Table 2:** Complications and secondary outcomes variables of patients.

	Helmet (n = 29)	Face mask (n = 30)	*P* value
**Cumulative complications**^a^	3 (10%)	13 (43%)	0.004
Claustrophobia	2 (7%)	0 (0%)	0.237
Skin lesions	1 (3%)	4 (13%)	0.353
Severe air leakage^b^	0 (0%)	4 (13%)	0.112
Eye irritation	0 (0%)	3 (10%)	0.237
Gastric distension	0 (0%)	1 (3%)	1.000
Bad tolerance	0 (0%)	1 (3%)	1.000
Duration of NIV, median (IQR) hours	6/(4–12)	6/(4–13)	0.802
ICU stay, median (IQR) days	7/(5–8)	8/(6–10)	0.100
Intubation rate	1 (3%)	3 (10%)	0.612
ICU mortality	1 (3%)	1 (3%)	1.000

*NIV* noninvasive ventilation, *ICU* intensive care unit, *IQR* interquartile range.

^a^None of the patients developed more than one complication.

^b^It was defined as air leakage too much to perform the NIV successfully.

### Effect of NIV with the helmet on secondary outcomes

PaO_2_/FiO_2_s were higher in the helmet group at 1 h, and at the end of NIV treatment compared with those in the face mask group (253.14 ± 64.74 mmHg vs 216.06 ± 43.86 mmHg, and 277.07 ± 84.89 mmHg vs 225.81 ± 63.64 mmHg, *P* = 0.013 and 0.012, respectively). Patients’ respiratory rates decreased obviously at 1 h, 4 h, and the end of NIV treatment in both groups, and the differences between the groups were statistically significant (18.10 ± 4.13 breaths/min vs 20.33 ± 2.54 breaths/min; 17.69 ± 3.09 breaths/min vs 20.07 ± 2.86 breaths/min; and 17.10 ± 2.97 breaths/minvs 19.30 ± 2.04 breaths/min; *P* = 0.016, *P* = 0.003, and *P* = 0.002, respectively). There were no significant effects on pH and partial pressure of arterial carbon dioxide (*P* > 0.05) (Table 3).

**Table 3:** Time courses and comparison of arterial blood gas and vital sign variables.

	NIV 1 h		NIV 4 h		The end of NIV	
	Helmet (n = 29)	Face mask (n = 30)	Helmet (n = 29)	Face mask (n = 30)	Helmet (n = 29)	Face mask (n = 30)
PaO_2_/FiO_2_	253.14 ± 64.74^a^^,^^b^	216.06 ± 43.86^a^	267.04 ± 86.73^a^	229.41 ± 75.10^a^	277.07 ± 84.89^a^^,^^b^	225.81 ± 63.64^a^
PaCO_2_ (mmHg)	38.79 ± 3.74	37.99 ± 4.56	38.87 ± 4.46	36.31 ± 5.05	38.76 ± 4.60	36.70 ± 5.08
Arterial pH	7.41 ± 0.05	7.43 ± 0.05	7.42 ± 0.03	7.45 ± 0.05	7.43 ± 0.04	7.45 ± 0.05
RR (breaths/min)	18.10 ± 4.13^a^^,^^b^	20.33 ± 2.54^a^	17.69 ± 3.09^a^^,^^b^	20.07 ± 2.86^a^	17.10 ± 2.97^a^^,^^b^	19.30 ± 2.04^a^
HR (breaths/min)	94.48 ± 19.34	92.50 ± 12.66	91.93 ± 17.46	90.53 ± 10.66	90.79 ± 17.73	89.50 ± 9.12
SBP (mmHg)	121.34 ± 15.08	129.73 ± 10.93	122.38 ± 15.76	129.83 ± 12.26	122.79 ± 15.11	130.47 ± 11.60

*PaO*_*2*_*/FiO*_*2*_ the ratio of partial pressure of oxygen in arterial blood to fraction of inspired oxygen, *NIV* noninvasive ventilation, *PaCO*_*2*_ arterial partial pressure of carbon dioxide, *RR* respiratory rate, *HR* heart rate, *SBP* systolic blood pressure.

^a^The differences of outcomes between at baseline and corresponding time point were statistically significant (*P* < 0.05).

^b^Compared with the face mask, the helmet significantly increased PaO_2_/FiO_2_ or decreased RR at corresponding time point.

Patients tolerated the helmet better than the face mask at 4 h (proportion of excellent tolerance: 83% (24/29) vs 47% (14/30), respectively; *P* = 0.004) and at the end of NIV treatment (proportion of excellent tolerance: 69% (20/29) vs 30% (9/30), respectively; *P* = 0.03); the difference at 1 h was not significant (*P* > 0.05) (Table 4). One patient in the face mask group had to remove the mask and stopped NIV because of poor tolerance.

**Table 4:** Patient’s tolerance at each time point during NIV treatment.

	NIV 1 h		NIV 4 h		End of NIV	
	Helmet (n = 29)	Face mask (n = 30)	Helmet (n = 29)	Face mask (n = 30)	Helmet (n = 29)	Face mask (n = 30)
Excellent	26 (90%)	21 (70%)	24 (83%)	14 (47%)^a^	20 (69%)	9 (30%)^a^
Good	3 (10%)	9 (30%)	3 (10%)	12 (40%)	6 (21%)	10 (33%)
Moderate	0 (0%)	0 (0%)	2 (7%)	4 (13%)	3 (10%)	10 (33%)
Bad	0 (0%)	0 (0%)	0 (0%)	0 (0%)	0 (0%)	1 (4%)

^a^The proportions of excellent tolerance at 4 h (*P* = 0.004) or the end of NIV treatment, (*P* = 0.03) were statistically significant between groups.

The rate of intubation was 3% (1/29) in the helmet group, which was slightly lower than the rate in the face mask group [3/30 (10%); *P* = 0.612]. There was no significant difference in the duration of NIV between the helmet and face mask group, respectively [median (IQR), 6 (4–12) h vs 6 (4–13) h; *P* = 0.802], or in the length of ICU stay [median (IQR), 7 (5–8) days vs 8 (6–10) days; *P* = 0.100] or ICU mortality between the two groups [3% (1/29) vs 3% (1/30); *P* = 1.000)] (Table 2).

## Discussion

The results indicated that NIV with a helmet decreased complications related to NIV, increased oxygenation, decreased respiratory rate, and improved tolerance compared with NIV with a face mask in patients with chest trauma, while there was no evidence indicating that NIV with a helmet shortened ICU stay, and reduced intubation rates and ICU mortality.

In the past several decades, invasive mechanical ventilation was the prioritized support to improve gas exchange and facilitate chest stabilization in patients with chest trauma-related ARF^35^. However, complications, such as ventilator-associated pneumonia and barotrauma, were associated with prolonged ventilation and even led to higher mortality rates^10,17,36^. With the introduction of NIV, Antonelli et al. reported similar effects regarding improved gas exchange and fewer complications compared with invasive mechanical ventilation in a mixed population including 12% of patients with trauma^17^. Subsequently, studies indicated that early use of NIV promoted lung recruitment and reduced intubation and mortality rates^3,11,12,37^. However, complications related to NIV were still common and most were associated with using a face mask. The complication accounted for as high as 14.8% of NIV failure and caused a three-fold increase in hospital mortality^19^. Recently, a new NIV interface, a helmet, has been used to reduce complications related to NIV and improve tolerance in patients with ARF caused by severe pneumonia, exacerbation of chronic obstructive pulmonary disease, and acute cardiogenic pulmonary edema^22,38^. However, the benefits of NIV with a helmet were uncertain in patients with ARF caused by chest trauma.

This trial indicated that using the helmet significantly reduced the incidence of complications related to NIV. Skin lesions are a frequent problem with long-term NIV with face masks and have even led to NIV failure^13,22^. The helmet is made of a transparent plastic hood, which does not touch the patient’s face, nose bridge, or eyes, and in our study, the inflatable collar of the helmet caused redness of the neck skin in only one patient and prevented eye irritation^38^. None of the three patients with mild facial trauma in the helmet group developed complications. Four patients in the control group developed facial and nasal bridge skin lesions. As a result, the helmet was a good choice of interface, especially for patients with mild facial trauma^31^. Furthermore, the soft collar in the helmet is designed to seal the neck and minimize air leaks. In this study, the helmet was associated with fewer air leaks and better comfort than the face mask. Eye irritation resulted from direct contact between the upper edge of the oronasal mask and air leakage around the nasal bridge. For patients with clavicular or rib fractures, we selected the helmet without armpit braces, which further improved comfort and reduced the incidence of complications. However, claustrophobia occurred in the helmet group, which might have originated from the completely closed structure. The closed structure interfered mildly with the transmission of sound and may have caused claustrophobia, especially in those who were easily anxious, as can be seen with trauma patients in a stressed state. Claustrophobia could be mitigated by proactive communication from medical staff. It is necessary to explore whether wearing an in-ear earphone could reduce the incidence of claustrophobia, in future research.

In this trial, NIV with a helmet improved PaO_2_/FiO_2_ and decreased respiratory rate compared with NIV with a face mask, suggesting that the helmet was effective in relieving dyspnea. This might be attributed to the unique advantages of the helmet. First, the helmet did not require interrupting NIV treatment when patients drank, communicated, and cleared sputum^31^. Continuous NIV is crucial in the early phases of respiratory insufficiency and might reduce the need for intubation^39^. Second, the helmet was better tolerated in patients with chest trauma; therefore, patients did not require conversion to an invasive ventilator^31^. Our results showed that 83% of the patients in the helmet group showed excellent tolerance compared with 47% of the patients in the face mask group after 4 h of NIV treatment. Moreover, the helmet could be fit to any patient regardless of differences in their facial contour, such as edentulous teeth, facial deformity, or trauma^22,40^. Overall, patients with chest trauma-related ARF responded favorably to NIV with a helmet because of the comfort, and better tolerance and oxygenation; thus, helmet use might broaden the use of NIV.

In this trial, the incidence of complications in the face mask group was higher than that reported in other patient populations^22,31,40^. Currently, there are limited data describing complications related to NIV in patients with chest trauma. The only study of patients with thoracic trauma involved only 16% of the recruited patients, and reported a complication rate related to face masks of 33%^26^. The higher rate of complications in our trial might be attributed to pain caused by trauma, and stress and traumatic psychological reactions of patients with trauma, which decreased patients’ ability to tolerate the mask^39^.

The duration of noninvasive ventilation was relatively short in this study, although the duration was similar to a few related studies of NIV with a helmet^21,23^. This lower duration is because our hospital is located in an industrial area where many highways converge and construction sites abound, and which leads to multiple types of accidents. However, patients could be admitted to the hospital in the early stages of trauma. Early application of NIV alleviated pulmonary edema and prevented its development, and shortened the duration of ventilatory support^18,41^. This study serves to broaden the indications for helmet use and provides a new option for patients with chest trauma in addition to NIV with a face mask^37^ and high-flow nasal cannula therapy^42,43^.

Our study had several limitations. First, given the nature of the interface, the attending physicians could not be blinded during the analysis, and this may have introduced performance bias^22^. The helmet is a new interface, which may have affected medical staff regarding judging clinical outcomes despite the fact that we tried to keep the staff blind and to have the patients managed without discrimination. Second, the limited sample size does not present enough evidence for helmet use to reduce NIV failure and intubation rates, shorten the length of ICU stay, and decrease ICU mortality. Caution is needed regarding evaluating the clinical benefits of a helmet except for the lower incidence of complications, higher PaO_2_/FiO_2_, and better tolerance; large RCTs aiming to determine the long-term prognosis are needed. Third, to reduce invasive procedures in patients with trauma, we did not introduce extravascular lung water monitoring techniques, which somewhat limited the demonstration of pulmonary edema and quantitative measurement of the pathophysiological changes; however, these changes can be partly qualitatively judged with lung computed tomography imaging. Fourth, this study was terminated before reaching the planned sample size because of the prespecified interim analysis, which might have led to potential bias and exaggerated findings, particularly for the small number of events^44^. However, another study considered that overestimation was acceptable if the proportion of events in the interim analysis was > 50%, as in this study^45^.

## Conclusions

NIV with a helmet decreased complications related to NIV, increased PaO_2_/FiO_2_, and improved tolerance compared with NIV with a face mask in patients with chest trauma. The new helmet may be a valid and optional interface for NIV in patients with chest trauma-related ARF.

## Data Availability

The data that underlie the findings of this trial are available from the corresponding authors, Q.L. or R.C.C., upon reasonable request.

## Abbreviations

NIV: Noninvasive ventilation
ARF: Acute respiratory failure
RCT: Randomized controlled trial
PaO_2_/FiO_2_: Arterial partial pressure of oxygen/fraction of inspired oxygen
ICU: Intensive care unit
PS: Pressure support
SpO_2_: Saturation of pulse oxygen
RR: Respiratory rate
